# Acid–Base and Electrolyte Balance Responses in the Performance of Female Ultramarathon Runners in a 45 km Mountain Race

**DOI:** 10.3390/nu17050751

**Published:** 2025-02-20

**Authors:** Marcelo Romanovitch Ribas, Danieli Isabel Romanovitch Ribas, Priscila Fernandes, Georgian Badicu, Elto Legnani, Anderson Caetano Paulo, Luca Paolo Ardigò, Júlio Cesar Bassan

**Affiliations:** 1 Postgraduate Program in Electrical Engineering and Industrial Informatics, Universidade Tecnológica Federal do Paraná, Curitiba 80230901, Brazil; mromanovitch@yahoo.com.br; 2 Human Genetics Laboratory, Centro Universitário Autônomo do Brasil (UniBrasil), Curitiba 82821020, Brazil; danieliribas@yahoo.com.br; 3 Postgraduate Program in Physical Education, Universidade Tecnológica Federal do Paraná, Curitiba 81310900, Brazil; prifer82@hotmail.com (P.F.); elto@utfpr.edu.br (E.L.); cpaulo@utfpr.edu.br (A.C.P.); jcbassan@utfpr.edu.br (J.C.B.); 4 Department of Physical Education and Special Motricity, Faculty of Physical Education and Mountain Sports, Transilvania University of Braşov, 500068 Braşov, Romania; 5 Department of Teacher Education, NLA University College, 0166 Oslo, Norway

**Keywords:** ultramarathon, electrolyte balance, acid–base imbalance, female athletes, performance

## Abstract

**Objectives/Background:** This study investigated the influence of acid–base and electrolyte balance on the performance of female athletes in a 45 km ultramarathon. The aim was to analyze the impact of these variables on performance, particularly in athletes with varying completion times. **Methods:** Nineteen female athletes (mean age: 35.9 ± 6.5 years) were divided into three groups based on their race completion times: faster, intermediate, and slower. Blood samples were collected before and after the race to assess biochemical variables and arterial blood gases. **Results:** Significant differences in potassium (K^+^) levels were found in the intermediate (*p* = 0.003, adjusted *p* = 0.01) and slower (*p* = 0.006, adjusted *p* = 0.03) groups. Hematocrit (Hct) showed a significant reduction in the intermediate group (*p* = 0.007, adjusted *p* = 0.04). In arterial blood gas variables, significant reductions in HCO_3_^−^ and pCO_2_ were observed in the faster (HCO_3_^−^: *p* = 0.002, adjusted *p* = 0.008; pCO_2_: *p* = 0.007, adjusted *p* = 0.02) and intermediate (HCO_3_^−^: *p* = 0.005, adjusted *p* = 0.02) groups. In the slower group, significant reductions in pH (*p* = 0.001, adjusted *p* = 0.004) and HCO_3_^−^ (*p* = 0.001, adjusted *p* = 0.004) were found. The correlation between post-race Na^+^ levels and performance was significant in the intermediate group (*p* = 0.01, adjusted *p* = 0.02). **Conclusions:** Acid–base and electrolyte imbalances significantly affect ultramarathon performance, with a greater impact observed in athletes with slower times. These findings highlight the importance of strategies to optimize electrolyte and acid–base balance in endurance events.

## 1. Introduction

Ultramarathons are races that exceed the standard marathon distance of 42.195 km, conducted across diverse terrains and surfaces under varying environmental conditions, which impose extreme challenges and demand significant physiological adaptations from the human body [1,2]. In recent years, these events have gained increasing popularity, particularly among professional female athletes [1,3,4], who now account for 20% of the total number of finishers. This increasing participation has highlighted the necessity to better understand the specific physiological demands of female ultramarathon runners [5].

Environmental conditions, such as temperature and altitude, significantly affect oxygen transport and physical performance [6]. According to Kelly [7], environmental conditions such as temperature and altitude have significant effects on oxygen transport and physical performance. In endurance events conducted at high altitudes, the reduction in the partial pressure of oxygen (pO2) may result in hypoxemia, directly impacting respiratory efficiency and the aerobic capacity of athletes. Furthermore, electrolyte imbalances, such as decreases in sodium and potassium levels, can adversely affect muscle function and contribute to fatigue [8]. These conditions substantially influence acid–base balance, ventilation, and aerobic performance, all of which are critical factors for maintaining performance during prolonged endurance events [7]. Research indicates that such imbalances may further exacerbate fatigue and hinder the body’s ability to perform at high levels, especially in extreme environments such as high altitudes and during prolonged exercise [8].

Compared with men, women demonstrate performance differences of 10–20% in long-duration races, achieving their fastest times between the ages of 35 and 45. This age range may shift upward as the distance or duration of the race increases [4]. Additionally, women exhibit distinct physiological responses, including greater preservation of body fat and differing immunological and hormonal responses [9,10]. These physiological differences may contribute to distinct responses to endurance stress, particularly in terms of metabolic efficiency, thermoregulation, and acid–base balance during ultramarathons [5].

The increasing interest in female participation in ultra-endurance events highlights the need to understand the unique physiological characteristics of female athletes. However, there is a scarcity of studies exploring these nuances [5]. Existing research [11,12] tends to focus on short- and middle-distance races, leaving a significant gap in the literature regarding physiological responses in women, who remain underrepresented in endurance sports research [4]. This gap is even more prominent when considering the impact of acid–base disturbances and electrolyte imbalances on performance, particularly in long-duration, high-intensity events such as ultramarathons [13].

Addressing these gaps, the present study aims to analyze the biochemical and blood gas responses of female ultramarathon runners, focusing on the correlation between electrolyte and acid–base balance and athletic performance during a 45 km mountain race. The investigation seeks to understand how acid–base equilibrium and electrolyte variables influence the performance of female ultramarathoners, thereby contributing to a deeper understanding of the specific physiological responses of women in ultra-endurance events.

## 2. Materials and Methods

### 2.1. Design

This cross-sectional study was conducted during the Ultramarathon Perdidos SkyMarathon^®^, a 45 km race held in the region of Tijucas do Sul, Paraná, at a significant altitude with a total elevation gain of 2630 m above sea level. The race began at 6:00 AM, with ambient temperatures starting at 13 °C and gradually rising to 19 °C throughout the day. Relative humidity ranged from 70% to 90%, creating a humid and challenging environment for thermoregulation and electrolyte balance. These environmental conditions were monitored using climatological data, as they can directly influence the physiological and biochemical responses of athletes during and after the race.

### 2.2. Participants

The sample size was calculated using G*Power software, version 3.1.9.2, for repeated measures ANOVA (within and between groups). The parameters included a significance level of 5% (α = 0.05), a statistical power of 80%, three groups, and two measurements (pre- and post-race). An effect size of (medium) was assumed based on similar previous studies investigating the physiological and biochemical responses of ultramarathon runners, including Hoppel et al. [14] and Kishi et al. [15]. Although the calculated sample size suggested a minimum of 42 participants to achieve adequate statistical power, the final sample included only 19 participants due to recruitment challenges. Consequently, the study’s power was reduced (~0.55), which is acknowledged as a limitation. Nevertheless, the sample size aligns with other exploratory studies in endurance sports and provides valuable insights into electrolyte responses and acid–base balance during ultramarathon events.

The inclusion criteria required athletes with 3–4 years of experience in mountain running and participation in at least two races exceeding 21 km and one exceeding 42 km, which were completed at least two years before data collection. Participants also needed to train 5–6 times per week, for 1–2 h per day and over 3 h on weekends, with no reported musculoskeletal disorders. Weekly training volume was assessed through a self-reported questionnaire. Athletes reported their average weekly training duration and intensity over the past 3 months prior to the study. The exclusion criteria included runners who did not sign the informed consent form, failed to complete one of the two data collection phases, or expressed a desire to withdraw consent. Additionally, participants were excluded if they presented (1) a history of metabolic disorders, (2) recent musculoskeletal injuries, (3) use of medications affecting fluid balance, (4) failure to meet the minimum weekly training requirements, or (5) non-compliance with the study protocol.

Of the 34 initial participants, 15 did not met one or more of these criteria and were excluded, resulting in a final sample of amateur athletes aged between 32 and 55 years. These criteria ensured sample homogeneity and minimized confounding factors. 

The athletes were categorized into three groups based on their running times for comparative analysis:

Group 1 (Fastest Times): times ranged from 473 to 552 min (*n* = 7).
Group 2 (Intermediate Times): times ranged from 563 to 617 min (*n* = 6).
Group 3 (Slowest Times): times ranged from 633 to 658 min (*n* = 6).

The study was approved by the Research Ethics Committee (REC) under approval number 2.275.040 on 14 September 2017, and adhered to Resolution 466/12 of the Brazilian National Health Council.

### 2.3. Instruments and Procedures

Data collection was conducted in two phases. The first phase took place one day before the event at the race venue, during the distribution of race kits (mandatory items and athlete identifiers). The second phase occurred immediately after the 45 km race. In both phases, anthropometric assessments and venous capillary blood samples were collected to determine the concentrations of electrochemical elements and acid–base disturbances, including hydrogen potential (pH), Na^+^ (sodium), K^+^ (potassium), Ca^2+^ (calcium), Glu (glucose), pCO_2_ (partial pressure of carbon dioxide), pO_2_ (partial pressure of oxygen), Lac (lactate), hematocrit (Hct), and HCO_3_^−^ (bicarbonate).

### 2.4. Anthropometric Assessment

The anthropometric evaluation consisted of measuring body weight (BW), height, body fat percentage (%F), body water (BWt, L), and lean mass (LM). BW was measured using a platform-type anthropometric scale (Filizola, São Paulo, Brazil) with a precision of 100 g, while height was determined with a portable stadiometer (Seca, Hamburg, Germany) with a precision of 0.1 cm, considering the arithmetic mean of three consecutive measurements as the final value. The body fat percentage (%F), body water (BWt, L), and lean mass (LM) were assessed using the whole-body tetrapolar bioelectrical impedance method (Maltron, Rayleigh, UK) at a frequency of 50 kHz [16].

### 2.5. Blood Collection

Venous blood samples with real-time access and analysis were collected using the minimally invasive capillary blood sampling technique through fingertip puncture [17]. This method was chosen due to its feasibility in a field-based ultramarathon setting, where venous sampling would be logistically challenging and more invasive for the athletes. The skin was sanitized with 70% alcohol before sampling to ensure sterility. Blood was collected using a heparin-treated 200 μL capillary tube (Capillary Tubes 250 Roche^®^) to prevent coagulation and ensure sample integrity.

Blood analysis was conducted using the GEM Premier 3000 gas analyzer (Instrumentation Laboratory, Bedford, MA, USA), which has been validated for accuracy in clinical and research settings. The analyzer assessed key parameters such as pH, Na^+^, K^+^, Ca^2+^, Glu, pCO_2_, pO_2_, Lac, Hct, and HCO_3_^−^, all of which are critical for evaluating acid–base disturbances [18].

To ensure data accuracy and reliability, all equipment was calibrated and validated before and during the study, following the manufacturer’s guidelines. Previous studies have demonstrated high correlation coefficients (0.91–0.99) between the GEM Premier 3000 and other reference analyzers, supporting its precision in biochemical analysis [19]. To minimize sample degradation and variability, blood samples were collected within approximately one minute after race completion and processed immediately. Results were obtained approximately 85 s after the sample was introduced into the blood gas analyzer, ensuring real-time biochemical assessment [19].

### 2.6. Statistical Analyses

The data were analyzed using R software, version 4.0.5, utilizing specific packages for different statistical procedures. Descriptive statistics (mean and standard deviation) were used to summarize the data, and Repeated Measures Analysis of Variance (ANOVA-RM) was performed to compare biochemical, blood gas, and body composition variables between pre- and post-race time points within each group and among groups categorized by race time. The car package was employed to verify ANOVA assumptions. To assess the magnitude of differences, effect size (Partial Eta^2^) was calculated using the effectsize package. Additionally, achieved statistical power (%) was reported using the pwr package to ensure the reliability of the findings. Pearson’s correlation coefficient (r) and simple linear regression were used to examine the relationship between weight loss and changes in electrolytes and hematocrit, with the coefficient of determination (R^2^) and statistical significance (*p*-values) included in the results. These analyses were conducted using the rstatix package. F-values from ANOVA-RM and adjusted *p*-values were reported to enhance the statistical interpretation. Scatter plots were generated using ggplot2 and ggpubr to visualize the relationships between biochemical variables and race time. The significance level adopted was *p* ≤ 0.05.

## 3. Results

The analysis of biochemical variables pre- and post-race (Table 1) revealed that sodium (Na^+^) levels showed no significant differences between pre- and post-race moments in any of the groups (*p* > 0.05), indicating the stability of this electrolyte. In contrast, potassium (K^+^) levels significantly decreased post-race across all groups, with high effect sizes (Partial Eta^2^ > 0.60). The greatest reduction was observed in the Intermediate Times group (*p* = 0.003, Partial Eta^2^ = 0.85), followed by the Slowest Times group (*p* = 0.006, Partial Eta^2^ = 0.76), and lastly, the Fastest Times group (*p* = 0.03, Partial Eta^2^ = 0.61). Glucose (Glu) levels did not show statistically significant variations between pre- and post-race moments (*p* > 0.05), although individual fluctuations were observed. Calcium (Ca^2+^) levels also remained unchanged across all three groups, with no significant impact (*p* > 0.05). Lactate (Lac^−^) levels showed a post-race increasing trend in all groups; however, the results did not reach statistical significance (*p* > 0.05). Nevertheless, the moderate effect observed in Partial Eta^2^ suggests that this increase may have physiological relevance.

Table 2 presents the relationship between weight loss (%) and changes in electrolytes and hematocrit following the 45 km mountain race. The results indicate that K^+^ and Hct% showed the strongest correlations with weight loss (r = 0.92, *p* = 0.25 and r = 0.95, *p* = 0.19, respectively), suggesting a possible effect of dehydration and hemodilution. Regression analysis revealed that weight loss accounted for 85% of the variation in K^+^ (R^2^ = 0.85) and 91% of the variation in Hct% (R^2^ = 0.91), further reinforcing this trend. On the other hand, Na+ did not show a strong relationship with weight loss (r = 0.70, *p* = 0.51; R^2^ = 0.48, *p* = 0.51), indicating that sodium homeostasis was maintained. Similarly, changes in body water (r = 0.65, *p* = 0.53) were not statistically significant.

**Figure 1:**
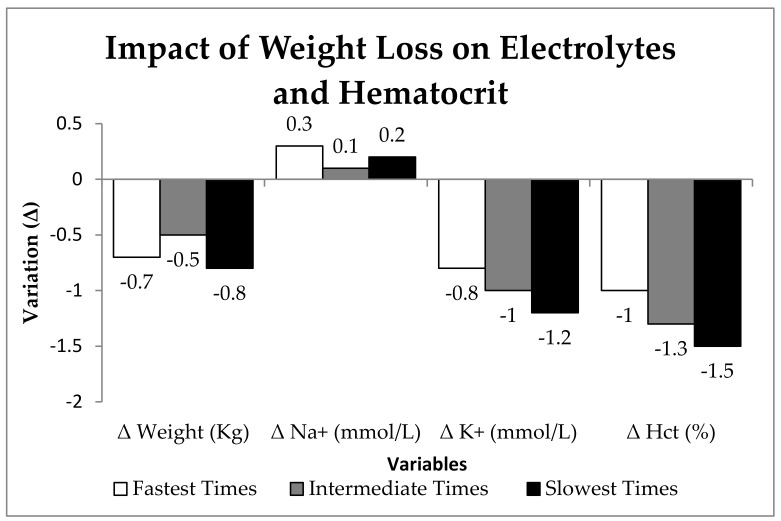
Relationship between weight loss and electrolyte/hematocrit variations in a 45 km mountain race.

Figure 1 illustrates the variation in body weight, electrolytes (Na^+^, K^+^), and hematocrit (Hct%) in mountain runners after a 45 km race, categorized according to race time (Fastest, Intermediate, and Slowest Times). The most pronounced reductions were observed in K^+^ and Hct%, particularly in the Intermediate and Slowest Times groups, suggesting a greater impact of dehydration in these athletes. In contrast, Na^+^ levels remained relatively stable, indicating possible sodium homeostasis regardless of race duration. Additionally, the Slowest Times group exhibited the greatest weight loss and Hct% reduction, reinforcing the influence of prolonged exertion on hemodilution. These findings suggest that the longer the race duration, the greater the variation in electrolytes and hematocrit, potentially associated with hydration status and physiological regulation.

The analysis of pre- and post-race blood gas parameters revealed significant reductions in HCO_3_^−^ and pCO_2_ across all groups, suggesting metabolic and ventilatory compensation. In the Fastest Times group, HCO_3_^−^ and pCO_2_ decreased significantly (*p* = 0.002; *p* = 0.007), with no relevant change in pH (*p* = 0.61). The Intermediate Times group showed a notable reduction in HCO_3_^−^ and pCO_2_ (*p* = 0.005; *p* = 0.04), with no significant changes in pH or pO_2_. The Slowest Times group exhibited the greatest reductions in pH, HCO_3_^−^, pCO_2_, and pO_2_ (*p* < 0.05), indicating a greater disruption in acid–base homeostasis. These findings suggest a progressive physiological adaptation as exercise duration increases.

**Figure 2:**
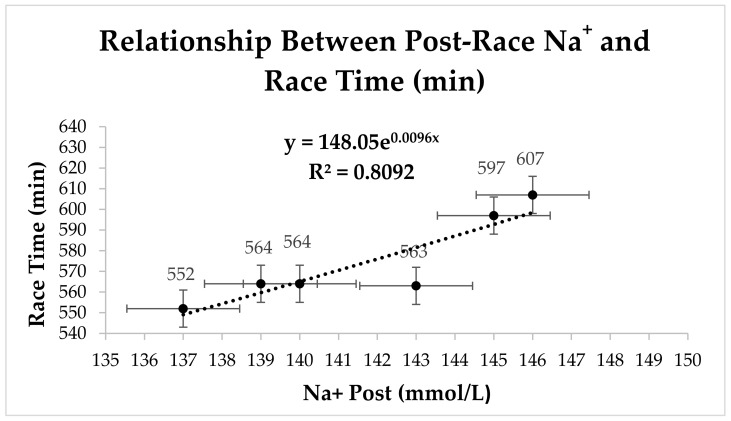
Relationship between post-race sodium levels (Na^+^) and race time in a 45 km mountain race.

Figure 2 illustrates the relationship between post-race sodium levels (Na^+^) and race time in endurance athletes. A positive exponential trend (R^2^ = 0.8092) is observed, indicating that higher Na^+^ concentrations are associated with longer race durations, possibly due to dehydration induced by prolonged exertion. Correlation analysis revealed that, for intermediate race times, post-race Na^+^ showed a significant correlation with race time (*p* = 0.01), suggesting that electrolyte balance plays a crucial role in athletic performance, particularly in intermediate endurance events.

**Figure 3:**
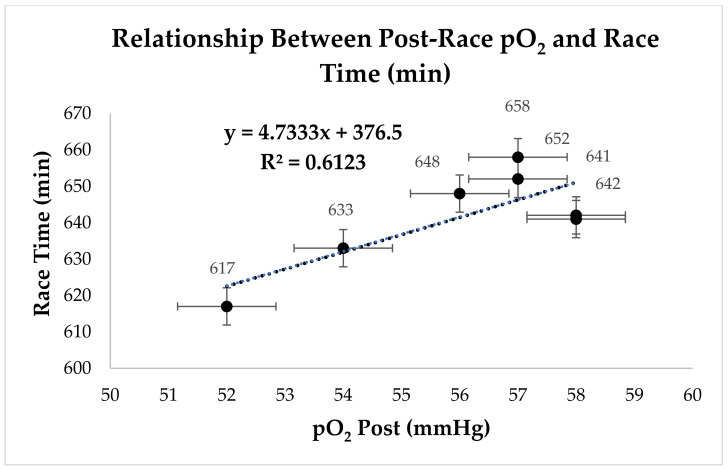
Relationship between post-race partial pressure of oxygen (pO_2_) and race time in a 45 km mountain race.

The correlation between race time and post-race partial pressure of oxygen (pO_2_) in the group with longer race times demonstrates a statistically significant relationship (*p* = 0.03, R^2^ = 0.6123). These findings highlight the importance of oxygenation in the performance of mountain ultramarathon runners, particularly for those with longer race times. As shown in Figure 3, as pO_2_ decreases, race time increases, suggesting a potential impact of ventilatory efficiency and oxygen transport on athletes’ endurance.

## 4. Discussion

This study aimed to determine the influence of acid–base and electrolyte balance on the performance of female athletes during a 45 km mountain ultramarathon. The hypotheses proposed in this study were partially confirmed. While post-race sodium (Na^+^) levels were expected to decrease significantly, particularly in athletes with longer race times, this trend was not observed. However, a significant correlation was found between post-race Na^+^ levels and race times in the intermediate group (*p* = 0.01, R^2^ = 0.8092), suggesting that sodium regulation plays a role in endurance performance.

In contrast, the hypothesis that partial pressure of oxygen (pO_2_) would significantly decrease in athletes with longer race times was confirmed (*p* = 0.03, R^2^ = 0.6123), reinforcing the idea that oxygenation limitations may impact performance under extreme fatigue conditions. Additionally, the findings support the hypothesis that prolonged exertion leads to significant changes in acid–base balance, as evidenced by the reductions in pH (*p* = 0.001, F = 27.92, Partial Eta^2^ = 0.82), HCO_3_^−^ (*p* = 0.001, F = 30.71, Partial Eta^2^ = 0.83), and pCO_2_ (*p* = 0.03, F = 7.19, Partial Eta^2^ = 0.54) in the slowest group. These results highlight the physiological adaptations associated with endurance exercise, emphasizing the interplay between electrolyte homeostasis, respiratory efficiency, and performance outcomes.

These alterations can be attributed to physiological mechanisms related to metabolic stress and the respiratory compensatory demand during prolonged exercise. The analysis of potassium revealed a marked decrease in the intermediate and longer-time groups (Table 1), consistent with the findings of Atanasovska et al. [20], Sumi et al. [21], and Juel [22]. Atanasovska et al. [20] investigated 11 healthy adults (9 men and 2 women) with a mean age of 30.55 years who were rowers, and identified post-exercise hypokalemia. This condition can compromise cardiac repolarization, potentially increasing the risk of arrhythmias and sudden cardiac death in susceptible individuals. Sumi et al. [21] demonstrated that metabolic acidosis can elevate extracellular K^+^, further hindering its reabsorption and exacerbating fatigue. Juel [22], in a review, also confirmed that the accumulation of extracellular K^+^ during exercise impairs muscle excitability.

During a 45 km race, muscles release potassium (K^+^) into the extracellular space, increasing its plasma concentration to maintain muscle excitability [23]. Post-exercise, muscles reabsorb this K^+^; however, when this process is incomplete, ionic imbalance can impair muscle contractions, contributing to fatigue. This condition reduces muscular efficiency, akin to an overloaded engine losing performance. Consequently, athletes with greater difficulty regulating K^+^ tend to record longer race completion times [20].

Regarding hematocrit, a reduction was observed in the intermediate and slowest groups, indicating hemodilution, a physiological adaptation to prolonged exercise. While hemodilution enhances blood flow and facilitates heat dissipation, it may compromise oxygen transport capacity, thereby affecting performance [23,24]. The variation in body weight and electrolyte balance observed in this study serves as an indirect indicator of the athletes’ hydration status. As shown in Table 2 and Figure 1, the reduction in potassium (K^+^) and hematocrit (Hct%) was associated with weight loss, suggesting the potential effects of dehydration and hemodilution [25]. Additionally, environmental factors such as temperature and humidity influence fluid and electrolyte loss, which can impact performance [26]. Changes in arterial blood gas parameters (Table 3) provide detailed insights into respiratory and acid–base responses following prolonged exertion [21]. Sumi et al. [21] demonstrated that, under normoxia, there was more pronounced metabolic acidosis during and after exercise, with a significant decrease in pH (*p* = 0.019) and HCO_3_^−^ (*p* = 0.001). This suggests that HCO_3_^−^ was utilized as a buffer to neutralize the increased hydrogen ions (H^+^) generated during exercise, an essential physiological response to minimize acidosis and maintain acid–base balance. These findings underscore the critical role of the buffering system in performance during intense activities.

Additionally, in the group with longer race times, the partial pressure of carbon dioxide (pCO_2_) also decreased (Table 3). Tiller [27] highlighted that marathons and ultramarathons temporarily reduce their pulmonary function due to respiratory muscle fatigue, leading to compensatory hyperventilation and a drop in pCO_2_ levels, which impairs ventilatory efficiency and performance. Similarly, the reduction in partial pressure of oxygen (pO_2_) observed in athletes with longer race times can be attributed to exercise-induced arterial hypoxemia (EIAH), commonly seen in athletes with high aerobic capacity [28]. EIAH occurs due to ventilatory inadequacy relative to high oxygen demands, reducing pO_2_ and oxygen saturation (SaO_2_) during prolonged exercise. This limits oxygen transport to active muscles, decreases VO2max, and compromises endurance performance [29].

The reduction in partial oxygen pressure (pO_2_) in athletes with longer race times suggests an impact on oxygen transport efficiency. Studies indicate that peak oxygen pulse (O_2_Ppeak), defined as VO_2_max/HRmax, is a key marker of aerobic capacity in endurance athletes, being closely associated with performance in prolonged efforts [30]. In the present study, the decline in pO_2_ among slower athletes may reflect ventilatory limitations and reduced oxygen transport efficiency, directly influencing endurance and performance in mountain ultramarathons.

Durand and Raberin [31] suggest that, although EIAH reduces SpO_2_, athletes with this condition may exhibit similar performance levels to those without EIAH under normoxic conditions. However, at moderate altitudes, EIAH becomes more pronounced, leading to a greater reduction in VO2max and aerobic performance. They also emphasize that EIAH may be underestimated in laboratory tests, as athletes encounter more severe conditions during real competitions.

The correlation between post-race Na^+^ levels and race time observed in the present study suggests a physiologically relevant mechanism for endurance performance. Sodium plays a crucial role in neuromuscular function and thermoregulation, making its maintenance essential during prolonged exertion. Fluid loss through sweating can increase plasma Na^+^ concentration, indicating dehydration, which may exacerbate fatigue and impair performance. Furthermore, inadequate sodium intake disrupts osmotic balance, compromising hydration status and cardiovascular efficiency. Thus, sodium homeostasis emerges as a key determinant of athletic performance, helping to prevent early fatigue and support post-exercise recovery [32,33].

Scatter plots were included to visualize the relationship between post-race Na^+^ levels and race time in Figure 2, as well as between post-race partial pressure of oxygen (pO_2_) and race time in Figure 3. These visualizations confirm a positive association between Na^+^ and race duration, supporting the hypothesis that sodium regulation plays a crucial role in endurance performance. The scatter plots also illustrate the variability in pO_2_ at longer race times, suggesting potential respiratory limitations under extreme fatigue conditions.

The balance between fluid and electrolyte intake is crucial. Both dehydration and overhydration without adequate sodium replacement can lead to hyponatremia, compromising performance [34]. Excessive fluid intake can dilute the blood, causing cerebral and pulmonary edema, and impairing aerobic performance. The decrease in pO_2_ observed in longer race times may indicate reduced respiratory efficiency or increased oxygen consumption, particularly under extreme fatigue conditions [21]. Tiller et al. [35] emphasize that EIAH, exacerbated by the alveolar–arterial O2 pressure gradient, is common in ultramarathons and can result in right ventricular dysfunction and pulmonary edema, further impairing oxygen diffusion and contributing to the decline in pO_2_.

Despite these significant findings, certain methodological limitations must be considered when interpreting the results. One of the main limitations of this study is the small sample size (*n* = 19), which resulted in reduced statistical power (~0.55). The sample size calculation, conducted using GPower 3.1.9.2, suggested a minimum of 42 participants to achieve adequate power (0.80). However, due to recruitment challenges, the final sample did not reach this threshold. This limitation increases the risk of type II errors and may reduce the generalizability of the findings. Future studies should aim for larger sample sizes to improve statistical robustness and validate these findings in a broader population of ultramarathon runners. **Although the study was conducted with 19 athletes, it is acknowledged that this sample size was not sufficient to ensure adequate statistical power, as indicated by the GPower calculation. This limitation has been explicitly recognized, and future studies should aim to include a larger sample to improve statistical robustness.** It is important to highlight that studies conducted in natural environments, such as ultramarathons, face logistical challenges that limit sample size. Nonetheless, the results provide valuable insights for future research. Another factor to consider is the lack of control over the intake of energy drinks containing caffeine, taurine, and guarana, which constitutes a limitation of the study, as these components can affect the observed results.

Future studies should incorporate more rigorous monitoring, such as controlled hydration protocols or real-time tracking of nutritional intake, to enhance data reliability. Additionally, the direct assessment of fluid intake and sweat rate during the race could provide a more precise differentiation between the effects of physical exertion and individual hydration strategies on electrolyte balance. Another factor to consider is the lack of control over dietary and fluid intake, which may have directly influenced the sodium, potassium, and glucose levels of the athletes. Variations in the nutritional strategies adopted by each participant could have introduced a source of variability in the biochemical results. Future studies with rigorous monitoring of nutrition and hydration during the race could provide a more precise analysis of these effects.

Additionally, individual factors such as menstrual cycles and hormonal variations were not controlled, which may have influenced the athletes’ electrolyte responses and physical performance. We acknowledge that hormonal fluctuations related to the menstrual cycle could have affected electrolyte balance and endurance performance. However, due to the logistical difficulties of collecting such data in a real-world ultramarathon event, it was not feasible to track menstrual cycle phases for all participants. Future studies should incorporate menstrual cycle tracking and hormonal profiling to assess the direct impact of these variations on physiological responses. While controlling these variables in field studies, particularly during ultramarathon events, is challenging, future research could incorporate menstrual cycle tracking and hormonal profiling to assess the direct impact of these variations on physiological responses and performance.

Furthermore, the participants in this study were amateur runners, which may limit the direct extrapolation of these findings to professional athletes. Elite athletes may exhibit different physiological adaptations and performance responses, requiring further research in this specific population.

Finally, the cross-sectional design employed, with data collection limited to pre- and post-race measurements, restricts the understanding of physiological changes occurring during the race and throughout the recovery period. Longitudinal studies that monitor athletes throughout the entire event and during post-race recovery could provide a more comprehensive and dynamic perspective on physiological responses to ultra-endurance efforts.

## 5. Conclusions

The results demonstrated that prolonged physical effort during the 45 km ultramarathon induced significant changes in biochemical variables and acid–base balance, particularly among athletes with longer race times. Athletes in these groups exhibited greater reductions in potassium (K^+^) levels in the Intermediate Times and Slowest Times groups, and in pH and HCO_3_^−^ in the Slowest Times group, indicating metabolic disturbances and increased physiological stress. In the Intermediate Times group, a significant correlation was also observed between post-race sodium (Na^+^) levels and race time. However, it is important to acknowledge that the study participants were amateur runners, which may limit the extrapolation of these findings to professional athletes. Additionally, the lack of strict control over dietary and hydration strategies, as well as the absence of menstrual cycle tracking, may have introduced variability in the biochemical responses observed. Despite these limitations, these findings underscore the importance of maintaining electrolyte and acid–base balance for performance in endurance events. Future research should aim to replicate these results in larger and more diverse populations, with controlled hydration and dietary intake, as well as tracking of hormonal fluctuations, to better understand their impact on ultramarathon performance. These insights highlight the need for training and nutritional strategies aimed at minimizing these impacts and improving performance in ultramarathons, particularly considering the metabolic demands of athletes with different competition levels and physiological profiles.

## Figures and Tables

**Table 1:** Comparison of pre- and post-mountain race biochemical variables across athlete groups classified by race time.

Fastest Times (*n* = 6)	Pre	Post	F	*p*-Value	Partial Eta^2^	Achieved Power (%)
Na^+^ (mmol/L)	141.8 ± 2.6	142.1 ± 1.5	0.04	0.84	0.008	5.03
K^+^ (mmol/L)	4.7 ± 0.4	3.9 ± 0.3	7.85	0.03 *	0.610	6.87
Glu (mg/dL)	101.5 ± 16.4	95.5 ± 36,0	0.37	0.56	0.06	5.24
Ca^2+^ (mmol/L)	1.2 ± 0.0	1.2 ± 0.0	2.14	0.20	0.30	5.98
Lac^−^ (mmol/L)	2.5 ± 0.8	3.5 ± 1.4	3.78	0.10	0.43	6.36
Hct (%)	45.3 ± 4.0	44.3 ± 2.9	0.30	0.60	0.05	5.20
Intermediate Times (*n* = 6)	Pre	Post	F	*p*-Value	Partial Eta^2^	Achieved Power (%)
Na^+^ (mmol/L)	142.0 ± 3.3	141.7 ± 3.6	0.04	0.84	0.008	5.03
K^+^ (mmol/L)	5.1 ± 0.9	4.0 ± 0.4	28.89	0.003 *	0.852	7.50
Glu (mg/dL)	86.3 ± 15.9	99.3 ± 12.2	1.47	0.27	0.228	5.76
Ca^2+^ (mmol/L)	1.3 ± 0.1	1.2 ± 0.0	1.53	0.27	0.234	5.78
Lac^−^ (mmol/L)	2.5 ± 0.8	3.0 ± 1.0	3.01	0.14	0.375	6.20
Hct (%)	43.8 ± 2.4	40.8 ± 2.1	19.28	0.007 *	0.794	7.35
Slowest Times (*n* = 7)	Pre	Post	F	*p*-Value	Partial Eta^2^	Achieved Power (%)
Na^+^ (mmol/L)	141.4 ± 2.9	142.1 ± 2.0	0.37	0.56	0.06	5.24
K^+^ (mmol/L)	5.1 ± 0.6	4.3 ± 0.5	16.29	0.006 *	0.76	7.28
Glu (mg/dL)	109.4 ± 37.1	95.1 ± 27.8	0.85	0.39	0.14	13.19
Ca^2+^ (mmol/L)	1.26 ± 0.08	1.19 ± 0.04	2.88	0.14	0.36	6.18
Lac^−^ (mmol/L)	2.6 ± 1.0	4.4 ± 2.0	4.02	0.09	0.44	6.41
Hct (%)	45.0 ± 2.9	42.7 ± 3.5	11.29	0.01 *	0.69	7.09

* *p* ≤ 0.05. K^+^ = potassium; Hct = hematocrit; mmol/L = millimole per liter.

**Table 2:** Effects of weight loss on electrolyte and hematocrit changes in a 45 km mountain race.

Variable	Fastest Times	Intermediate Times	Slowest Times	Pearson r	*p*-Value	R^2^	*p*-Value
Pre-race weight (kg)	56.7 ± 8.6	55.4 ± 6.9	58.1 ± 6.2	0.70	0.51	0.48	0.51
Post-race weight (kg)	56.0 ± 8.8	55.5 ± 7.1	57.7 ± 6.4	0.72	0.49	0.50	0.48
Δ Weight (kg)	−0.7 ± 0.5	0.1 ± 0.4	−0.4 ± 0.3	0.70	0.51	0.48	0.51
Body water (L)	−0.1 ± 0.2	−0.2 ± 0.3	−0.3 ± 0.4	0.65	0.53	0.42	0.54
Na^+^ (mmol/L)	0.3 ± 0.3	0.2 ± 0.2	0.1 ± 0.3	0.70	0.51	0.48	0.51
K^+^ (mmol/L)	−0.8 ± 0.6	−0.6 ± 0.5	−1.2 ± 0.7	0.92	0.25	0.85	0.25
Hct (%)	−1.0 ± 0.9	−1.2 ± 0.8	−1.5 ± 1.0	0.95	0.19	0.91	0.19

**Table 3:** Comparison of pre- and post-mountain race blood gas variables among athlete groups classified by race time.

Fastest Times (*n* = 6)	Pre	Post	Reference Values	F	*p*-Value	Partial Eta^2^	Achieved Power (%)
pH	7.40 ± 0.10	7.42 ± 0.05	7.30–7.40	0.28	0.61	0.05	5.19
HCO_3_**^−^** (mmol/L)	25.6 ± 3.1	22.1 ± 3.4	21–28	29.66	0.002 †	0.85	7.51
pCO_2_(mmHg)	37.6 ± 6.0	34.0 ± 7.0	41–51	18.90	0.007 †	0.79	734
pO_2_ (mmHg)	63.7 ± 6.3	59.1 ± 10	40–50	2.95	0.14	0.37	6.19
Intermediate Times (*n* = 6)	Pre	Post	Reference Values	F	*p*-Value	Partial Eta^2^	Achieved Power (%)
pH	7.41 ± 0.04	7.40 ± 0.02	7.30–7.40	0.17	0.69	0.03	8.99
HCO_3_^−^ (mmol/L)	26.2 ± 2.3	21.2 ± 3.4	21–28	21.70	0.005 †	0.81	86.72
pCO_2_(mmHg)	41.2 ± 4.8	33.8 ± 5.5	41–51	7.15	0.04 †	0.58	73.43
pO_2_ (mmHg)	60.2 ± 8.2	61.3 ± 6.9	40–50	0.08	0.77	0.01	5.79
Slowest Times (*n* = 7)	Pre	Post	Reference Values	F	*p*-Value	Partial Eta^2^	Achieved Power (%)
pH	7.44 ± 0.02	7.39 ± 0.02	7.30–7.40	27.92	0.001 †	0.82	7.31
HCO_3_^−^ (mmol/L)	24.9 ± 2.6	20.1 ± 3.0	21–28	30.71	0.001 †	0.83	7.34
pCO_2_(mmHg)	39.9 ± 3.1	33.0 ± 4.0	41–51	7.19	0.03 †	0.54	6.61
pO_2_ (mmHg)	66.7 ± 10.1	56.0 ± 2.2	40–50	10.68	0.01 †	0.64	6.86

† *p* ≤ 0.05. HCO_3_^−^ = bicarbonate; pCO_2_ = partial pressure of carbon dioxide; pO_2_ = partial pressure of oxygen; mmol/L = millimole per liter; mmHg = millimeters of mercury.

## Data Availability

The data are not publicly available due to ethical and privacy restrictions imposed by the Human Research Ethics Committee. Participants’ personal and sensitive information must be protected, and data sharing is restricted to ensure confidentiality.
